# Assessment of Winery By-Products as Ingredients as a Base of “3S” (Safe, Salubrious, and Sustainable) Fermented Beverages Rich in Bioactive Anthocyanins

**DOI:** 10.3390/foods14142514

**Published:** 2025-07-17

**Authors:** Berta María Cánovas, Irene Pérez-Novas, Cristina García-Viguera, Raúl Domínguez-Perles, Sonia Medina

**Affiliations:** Laboratorio de Fitoquímica y Alimentos Saludables (LabFAS), CSIC, CEBAS, Campus Universitario de Espinardo, Edificio 25, 30100 Murcia, Spain; bmcanovas@cebas.csic.es (B.M.C.); iperez@cebas.csic.es (I.P.-N.); cgviguera@cebas.csic.es (C.G.-V.); smescudero@cebas.csic.es (S.M.)

**Keywords:** oenological residues, kombucha, fermentation, bioactive phytochemicals, postbiotics, colour, healthy drinks, radical scavenging, reducing power

## Abstract

Oenological residues may cause environmental pollution when processing does not significantly reduce volume and/or harmful conditions. The lack of proper valorisation alternatives entails high disposal costs and resource inefficiency that jeopardise the sustainability and competitiveness of the industry. Interestingly, wine by-products are underappreciated sources of multipurpose bioactive compounds, such as anthocyanins, associated with health benefits. Alternatively, transforming oenological by-products into valuable co-products will promote sustainability and thus, create new business opportunities. In this context, the present study has assessed the applicability of winery by-products (grape pomace and wine lees) as ingredients to develop new functional kombucha-analogous beverages “3S” (safe, salubrious, and sustainable) by the Symbiotic Culture of Bacteria and Yeast (SCOBY). Concerning the main results, during the kombucha’s development, the fermentation reactions modified the physicochemical parameters of the beverages, namely pH, total soluble solids, acetic acid, ethanol, and sugars, which remained stable throughout the monitored shelf-life period considered (21 days). The fermented beverages obtained exhibited high anthocyanin concentration, especially when using wine lees as an ingredient (up to 5.60 mg/L at the end of the aerobic fermentation period (10 days)) compared with the alternative beverages produced using grape pomace (1.69 mg/L). These findings demonstrated that using winery by-products for the development of new “3S” fermented beverages would provide a dietary source of bioactive compounds (mainly anthocyanins), further supporting new valorisation chances and thus contributing to the competitiveness and sustainability of the winery industries. This study opens a new avenue for cross-industry innovation, merging fermentation traditions with a new eco-friendly production of functional beverages that contribute to transforming oenological residues into valuable co-products.

## 1. Introduction

The fermentation process is considered one of the most time-honoured food technologies for producing foods and foodstuffs. It is defined as a slow and controlled microbial process that provides a wide variety of health-promoting compounds, including prebiotics (substrates for microbial growth) and postbiotics (molecules resulting from microbial metabolism) [1,2]. Given the growing awareness of the relationship between diet and health, the ongoing interest in designing healthy diets has focused attention on these functional products, which include approximately 5000 foods worldwide, such as yoghurt, kefir, beer, kimchi, or kombucha [3].

Traditional kombucha is a beverage of Asian origin made by fermenting green or black tea in a sugary medium, promoted by a consortium of yeast and bacteria (acetic acid bacteria (AAB) and, to a lesser extent, lactic acid bacteria (LAB)) known by the acronym SCOBY (Symbiotic Culture of Bacteria and Yeast) [4,5]. Kombucha is a slightly acidic and carbonated beverage that, despite fermentation, is not classified as an alcoholic beverage as the alcoholic content is less than 1.2% (*v*/*v*) [6]. During fermentation, in kombucha, several bioactive compounds are generated, which can originate from both the vegetable matrix and the metabolic activity of the microorganisms. The set of phytochemical compounds present in these fermented drinks has been associated with a range of biological activities, namely antioxidant, antihyperglycemic, and antihyperlipidemic properties, among others [7]. Beyond green and black tea, the lack of a specific regulation allows for the production of kombucha using additional plant materials. As a result, to date, the use of alternative plant matrices, including broccoli, pitahaya, strawberry, cherries, and grapes, has been explored for the production of different types of kombucha with modified profiles of bioactive compounds, allowing for the attainment of alternative functional properties and bioactivities [2,4,8].

As part of our search for kombucha-like fermented drinks, using by-products from the agro-food industry has been signalled as a promising approach from a twofold perspective. First, it will contribute to the real valorisation of procedures towards new added value and market-demanded co-products, and second, it will enable the production of beverages with a diversified phytochemical profile compared to traditional performances. Both of them will promote the sustainability and competitiveness of this sector [9]. In this regard, wine production generates significant amounts of by-products that represent up to 30% of total grapes vinified, and whose disposal is costly due to their carbon content and low pH [10,11]. These by-products mainly include vine stems, grape skins, seeds, grape pomace, and wine lees. Among them, grape pomace, a mixture of grape skins and seeds resulting from the maceration phase, is the most widely assessed and used oenological by-product [12,13]. The potential of this by-product for kombucha brewing has been previously evaluated, describing enhanced concentrations of total (poly)phenols and anthocyanins in the beverages [9,11]. On the other hand, wine lees are mostly composed of intact or partially degraded yeasts, providing a source of nitrogen and macromolecules essential for the proper metabolic activity of SCOBY [12,14] and to stimulate the growth of probiotic microorganisms, such as LAB (e.g., *Oenococcus oeni* and *Lactiplantibacillus plantarum*) [12,14,15]. Thus, the presence of high concentrations of grape-featuring bioactive phenolics responsible for the health-promoting properties in these residues (mostly anthocyanins) has raised interest in reuse alternatives as a functional ingredient. According to this, the valorisation of both oenological by-products for the production of kombucha-like fermented beverages would be a valuable alternative due to their high concentration of bioactive (poly)phenols [16], among which anthocyanins stand out because of their proven antioxidant properties and contribution to obtaining beverages with a natural attractive colour, due to their shades ranging from reddish to bluish [17].

The present study aimed to uncover the potential of valorising winery by-products to obtain “3S” (safe, salubrious, and sustainable) kombucha-like fermented beverages, rich in bioactive anthocyanins.

## 2. Materials and Methods

### 2.1. Chemicals and Reagents

The standard phenolic compound cyanidin-3-*O*-glucoside chloride was obtained from TransMIT (Geiben, Germany). Formic acid, methanol, acetonitrile, and the disodium salt of ethylenediaminetetraacetic acid 2-hydrate (EDTA) were purchased from Panreac (Barcelona, Spain). Milli-Q water (Milli-Q system, Millipore, Bedford, MA, USA) was used for the preparation of reagents. All LC-MS grade solvents were purchased from JT Baker (Phillipsburg, NJ, USA). All kits for the determination of glucose, fructose, sucrose, ethanol, and acetic acid, as well as calibrators for sugars and acids, commercial controls for ethanol, system liquid, and wash solution, were supplied by BioSystems S.A. (Barcelona, Spain). Reagents and instrumental solutions were prepared according to the manufacturer’s instructions.

The reagents 2,2′-azino-bis(3-ethylbenzothiazoline-6-sulphonic acid) (ABTS), manganese dioxide, standard 6-hydroxy-2,5,7,8-tetramethylchroman-2-carboxylic acid (Trolox), 2,4,6-tris(2-pyridyl-s-triazine) (TPTZ), ferric chloride, and 2,2′-azobis(2-methylpropionamidine)dihydrochloride (AAPH) were purchased from Sigma-Aldrich (Steinheim, Germany). Sodium hydroxide, hydrochloric acid (37%), anhydrous disodium phosphate, and sodium bisphosphate were obtained from Panreac Química SA (Castellar del Vallés, Barcelona, Spain). Anhydrous sodium acetate and fluorescein were purchased from Fluka (Neu-Ulm, Germany), and glacial acetic acid was obtained from Carlo Erba Reactives (Milan, Italy).

### 2.2. Plant Material Selection, Processing, and Beverage Development

**Figure 1:**
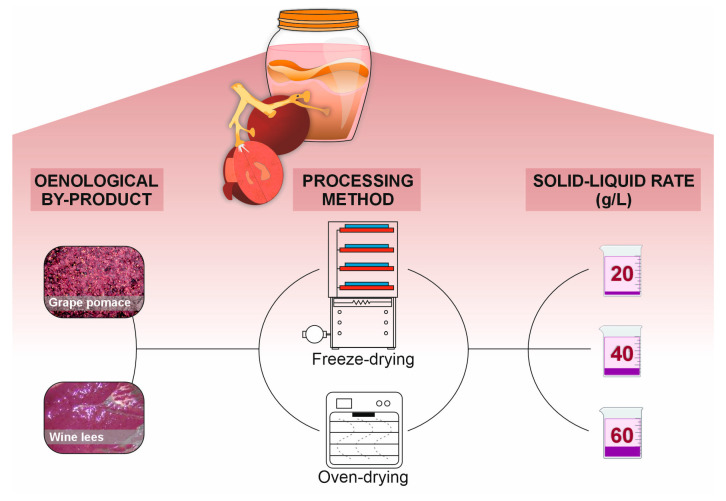
Experimental design including oenological residues, processing methods, and residue/water rate used in preparation of winery by-products infusions.

The winery by-products, grape pomace, and wine lees were obtained from the production of organic wine with grapes (*Vitis vinifera* L. var. “Monastrell”) from the Bodega Viña Elena (Jumilla, Murcia, Spain), harvest 2023. To determine the processing method (freeze-drying and oven-drying) and infusion rate (*w*/*v*, by-product/water) for the performance of the kombucha-like beverage with wine lees and grape pomace, these residues were dried to constant weight (at 40 °C for 72 h) and freeze-dried using a CHRIST 2-4D vacuum concentrator (Wolflabs, York, UK). Then, the dehydrated materials were ground to a fine powder for the preparation of infusions. The concentration of by-products used during the performance of the infusions was based on previous descriptions by Balmaseda et al. [9], with minor modifications due to the dehydration process applied in the present study. In this regard, given the moisture content of winery by-products (up to 80.0%), the weight/volume ratio used in the development of the beverage was monitored at three different concentrations (20, 40, and 60 g/L) to identify the best condition. After that, infusions were developed at ~90 °C for 15 min, in triplicate (Figure 1), and assessed on the capacity of the oenological residue to release the target bioactive anthocyanins.

The efficiency of the anthocyanin extraction was represented by their concentration in the beverage, expressed in milligrams per kilogram of by-product dry weight (mg/kg dw).

The kombucha-analogue fermented beverages were developed by infusing different percentages of residue in water (*w*/*v*) in triplicate (*n* = 3) (Figure 1). Specifically, freeze-dried wine lees or grape pomace were infused at a concentration of 20 g/L in 2 L of mineral water (KWL and KGP, respectively). Infusions were prepared at approximately 90 °C for 15 min and filtered through gauze with a 0.5 mm pore size to remove the plant material. Afterwards, sugar (sucrose) was added at a concentration of 70 g/L. Concurrently, a control beverage was prepared without adding by-products and with the same sucrose concentration (KC). The infusions were left to cool at room temperature (RT, 25 ± 2 °C), and a commercial SCOBY (Kefiralia-Burumart Commerce SL, Arrasate, Spain) was added to each one. Subsequently, samples corresponding to day 0 were collected from each of the beverages. The kombucha-like drinks were covered and left to ferment for 10 days at RT in the dark, and samples were collected every 2 days. At the end of the fermentation period (10 days), to monitor shelf-life, the beverages developed were packaged and stored at 4 °C for 21 days, with samples collected every 7 days. On each sampling day, acidity, total soluble solids (TSS), and pH were measured. For the remaining determinations, the samples were filtered through 0.22 µm PVDF filters (Millipore, MA, USA) and stored at −20 °C.

### 2.3. Physicochemical Parameters

As quality indicators, pH, TSS, sucrose, glucose, fructose, ethanol, and acetic acid were determined. Samples were analysed in triplicate (*n* = 3). For the analysis of pH and TSS, methodologies previously described by Salar et al. were applied [18]. The concentrations of glucose, fructose, sucrose, ethanol, and acetic acid were analysed using enzymatic methods using a Y15 Automatic Biochemistry Analyser (Code 83106, BioSystems, Barcelona, Spain).

### 2.4. Microbiological Tests

Microbiological analysis was carried out by Dr. Paula M. Periago at the Polytechnic University of Cartagena (UPCT, Department of Food Engineering and Agronomic Equipments), Murcia, Spain. Samples were analysed for *Salmonella* spp., *Listeria monocytogenes*, *Escherichia coli*, *Staphylococcus aureus*, and Enterobacteriaceae at the end of fermentation (day 10) and shelf-life (day 21) in triplicate (*n* = 3). For these analyses, samples were subjected to dilution with buffered peptone water (PW) (Scharlab, Barcelona, Spain) and homogenisation in a Stomacher, following the methodology described by Baenas et al. [19] and Salar et al. [18]. Microbial counts were expressed as colony-forming units per millilitre (CFU/mL).

### 2.5. Qualitative and Quantitative Analysis of Anthocyanins

The identification and quantification of anthocyanins were performed by HPLC-DAD-ESI/MSn and HPLC-DAD, respectively, according to the methods described by Salar et al. [20]. Anthocyanins were quantified as cyanidin 3-*O*-glucoside at 520 nm. The results were expressed as total anthocyanin content calculated as the sum of the concentrations of individual compounds and presented as milligrams per kilogram of dry weight (mg/kg dw) in infusions, and as milligrams per litre of kombucha-like beverage (mg/L).

### 2.6. Colour Analysis

Colour was determined by the CIEL*a*b* system using a Konica Minolta CR-5 Chroma Meter (Osaka, Japan) and a 2 mm cuvette by transmittance, concerning a light source set on D65 and a visual angle of 10°, according to previous descriptions in the literature [18].

### 2.7. Radical Scavenging Capacity and Reducing Power

The potential contribution of the newly developed beverages to the redox balance in living organisms was evaluated using three complementary methods. The ABTS and FRAP assays were performed according to the methodology described by Mena et al. [21] by measuring the variation in absorbance at 414 and 593 nm after 50 and 40 min of reaction, respectively. The ORAC assay was performed according to the methodology described by Migues et al. [22], in which the antioxidant activity is determined by two-hour kinetics by measuring the fluorescence intensity every 5 min at excitation and emission wavelengths of 485 and 520 nm, respectively.

For all three assays, 96-well microplates (Nunc, Roskilde, Denmark) and an Infinite M200 microplate reader (Tecan, Grödig, Austria) were used. The results of the ABTS+, FRAP, and ORAC assays were expressed as millimoles of Trolox equivalent per litre (mmol TE/L).

### 2.8. Statistical Analysis

The results were expressed as mean ± standard deviation (SD) or with the indication of the Least Significant Difference (LSD) as dispersion parameters (*n* = 3). SPSS 29.0 (LEAD Technologies, Inc., Chicago, IL, USA) was used for statistical univariate analysis and data processing. A homogeneity test and analysis of variance (ANOVA) were performed to identify significant differences between samples. Tukey’s multiple range test was applied as a post hoc categorisation test. The level of statistical significance was set at *p* < 0.05. Pearson’s correlation analyses were developed to set up associations between parameters. For multivariate data analysis, Principal Component Analysis (PCA) and a heatmap were obtained using MetaboAnalyst 6.0, freely accessible at https://www.metaboanalyst.ca (accessed on 21 April 2025).

## 3. Results and Discussion

### 3.1. Infusion Design as a Basis for Developing Fermented Beverages

The analysis of the oenological infusions (prepared previously to fermentation) revealed that the highest anthocyanin concentration is obtained by infusing freeze-dried materials, regardless of the specific residue considered (Table 1). In addition, comparatively, wine lees released higher amounts of anthocyanins into the beverage matrix, irrespective of the applied treatment (Table 1).

Once the processing method was selected, the optimal percentage of plant material for kombucha-like beverage performance was assessed by evaluating a range of performance rates. In this regard, the infusions made with freeze-dried wine lees, the highest efficiency for anthocyanin extraction during the infusion performance, corresponded to using 20 and 40 g/L (380.28 mg/kg dw, on average), which surpassed the yield obtained when applying 60 g/L by 26.0%. Alternatively, for grape pomace, the most efficient proportion of by-product per infusion volume when using freeze-dried material was 20 g/L, which provided significantly higher anthocyanin extraction than 40 and 60 g/L (by 24.7% and 37.4%, respectively) (Table 1). Furthermore, the highest proportions (solid–liquid rates) constituted a constraint for the practical development of the infusions due to the matrix density. According to these results, 20 g/L concentration was selected for both residues to simplify the elaboration protocol and obtain comparable samples. Although several individual anthocyanins were identified (delphinidin 3-*O*-glucoside, petunidin 3-*O*-glucoside, peonidin 3-*O*-glucoside, and malvidin 3-*O*-glucoside), malvidin 3-*O*-glucoside was, in most cases, the only anthocyanin at a quantifiable concentration.

This fact is in good agreement with the previously demonstrated enhanced preservation of bioactive compounds (e.g., (poly)phenols) by lyophilisation, in comparison with the application of thermal dehydration procedures (e.g., oven-drying) [10,23] because of their broadly reported thermolability [24]. In this sense, the results retrieved agree with previous descriptions of the anthocyanins’ stability in matrices such as grape skins, which support freeze-drying as the most effective dehydration method, in terms of anthocyanins preservation, compared to traditional oven-drying [25]. In some studies, oven-dried grape skin residues were reduced by up to 52% compared to freeze-dried samples [25].

### 3.2. Physicochemical Parameters

To shed light on the quality of the newly developed beverages, several parameters associated with the kombucha performance were monitored, namely pH, TSS, ethanol, and acetic acid (Table 2).

Hence, the kombucha control (KC) had a significantly higher initial pH value, surpassing the starting point of the wine lees and grape pomace-based kombucha-like beverages (KWL and KGP, respectively) by 43.8%, on average (Table 2). However, throughout the fermentation process, the pH decreased slightly (but significantly) in all beverages by an average percentage of 16.6%, which could affect the growth of fermenting microorganisms such as AAB and yeasts. Most AAB species can grow at pH ranging from 3.5 to 8.5, and some species have been reported to grow below pH 3 [26]. Alternatively, the optimum pH for yeast’s growth lies between pH 4.5 and 6.0 [27]. Nevertheless, during fermentation, the pH may drop to approximately 3.0, which could slow the metabolic activity of microorganisms but enhance the product’s preservation and acidity. Afterwards, throughout the storage period considered to evaluate shelf-life, the pH levels of all beverages remained almost unaltered in comparison with the level achieved at the end of the fermentation process (Table 2). These values were within the optimal range for human consumption (2.5–4.2), within a range typically associated with this type of beverage [2,11,28].

**Figure 2:**
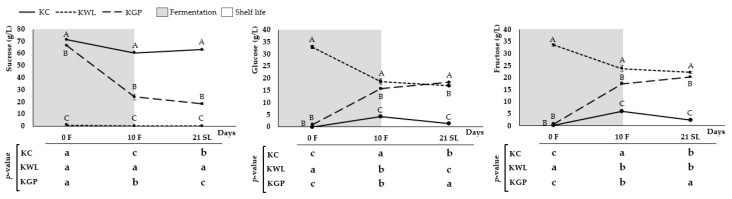
Sugar content of fermented beverages at days 0 and 10 of fermentation and after 21 days of storage (shelf-life). Distinct lowercase letters indicate significant differences between fermentation days and shelf-life for same beverage, and distinct capital letters designate significant differences between beverages on same day. Statistical differences were retrieved by resorting to one-way analysis of variance (ANOVA) and Tukey’s multiple range test at significance level of *p* < 0.05. F, fermentation; SL, shelf-life; KC, kombucha control; KWL, kombucha wine less; KGP, kombucha grape pomace.

Regarding the TSS (°Brix), this parameter varied minimally throughout fermentation and storage as beverages resulting from these processes remained within a tight range (6.05–7.04 °Brix) (Table 2). This evolution could be due to TSS mainly being influenced by the percentage of sugar added, even more than by the amount of vegetable matter incorporated [8]. Similarly, during fermentation, the yeast populations in the SCOBY would hydrolyse the added sucrose into the monosaccharides glucose and fructose [9]. Indeed, sucrose was completely hydrolysed at the beginning of the fermentation process in KWL, as can be seen in Figure 2, which could be due to the enzymatic machinery of the yeasts carried by this by-product, since these microorganisms can undergo autolysis processes at high temperatures, such as those reached during infusion [24]. Thus, the rupture of the yeast cell membranes during autolysis enables the release of endogenous hydrolytic enzymes, which could favour the hydrolysis processes [29]. As a result, the initial glucose and fructose content of this beverage increased and are available to be used as substrates in the different metabolic pathways of yeasts and bacteria that make up the SCOBY, then decreased throughout fermentation [30]. Concerning KC and KGP beverages, the sucrose hydrolysis process was more gradual, as the yeasts present in the SCOBY need to adapt to the medium before starting carbohydrate assimilation. KC exhibited a lower metabolism of these sugars during fermentation, evidencing that the SCOBY also requires sources of specific compounds and cofactors present in the vegetable matrix, such as nitrogen, for its proper functioning [31].

The increased glucose content of the by-product-based beverages is metabolised by yeasts towards ethanol production through the process of glycolysis [11]. KWL had a significantly higher ethanol content at the end of fermentation (5593.33 mg/L), exhibiting similar values to those previously obtained by Ayed et al. in grape juice kombucha after 10 days of fermentation [32]. This phenomenon is especially relevant for KWL because of the high specific microbiological profile of wine lees [33], which is in good agreement with previous reports showing that yeasts and bacteria resident in wine lees can contribute to the metabolic activity of *Saccharomyces* strains present in the SCOBY, responsible for the reproducibility of kombuchas [30]. On the other hand, and also related to the different microbiological profile of the separate oenological by-products, the synthesis of ethanol in KGP was lower than that recorded in KWL, reaching values of 1363.50 mg/L at the end of the fermentation stage. The concentration achieved remained stable throughout the shelf-life for KGP samples. Interestingly, a decreased ethanol content was recorded in KC and KWL beverages after storage at 4 °C. Since both of them differed from KWL only in the plant material used in beverage development, it could be hypothesised that enhanced alcohol production is ongoing in KWL by the metabolic activity of native yeasts and bacteria supplied by wine lees that could be responsible for a higher and sustainable alcohol content [34]. Nevertheless, it is worth mentioning from a marketable point of view that the alcohol content of all beverages was below the limits for alcoholic drinks stipulated by the European Union (1.2% *v*/*v*, equivalent to 9468 mg/L of ethanol) [6].

As a result of sugar and ethanol metabolism by AAB, there was a significant increase in the acetic acid content of all beverages during fermentation, especially those made from wine by-products [35]. As expected, due to the higher concentration of the referred substrates, KWL displayed a significantly higher acetic acid content at the end of fermentation (5.73 g/L) that surpassed KGP by 55.5% (Table 2). The acetic acid content remained stable and within the range previously observed in kombuchas made from wine by-products [9,11,35] throughout the shelf-life of all kombucha-like beverages. The acetic acid content of these fermented drinks can be influenced by various factors such as pH, day of fermentation, anaerobiosis caused by the growth of the cellulose mass, or even by the addition of a new yeast strain, for instance, in the beverages developed by adding wine lees as an ingredient [16,36]. However, despite the lack of a regulatory framework for this type of fermented beverage, the only specific legislation available to date from the Brazilian Government determines acceptable volatile acidity levels from 30 to 130 mEq/L (1.80 and 7.81 g/L of acetic acid, respectively) [28]. In line with this recommendation, our [11] results (5.73 and 2.55 g/L for KWL and KGP, respectively) are within the referred range and in agreement with Czarnowska-Kujawska et al. and Balmaseda et al. (5.04 and 3.10 g/L, correspondingly) [9,37], which has been associated with beverages with a pleasant taste and general consumer acceptance. On the contrary, a recent study on grape pomace-based kombucha displayed high acetic acid levels (up to 13 g/L) after 10 days of fermentation [11]. This discrepancy could be due to the characteristics of the by-product used in the kombucha development or differences in the fermentation process carried out.

### 3.3. Microbiological Tests

The assessment of the microbiological safety of the beverages developed at the end of the fermentation process and shelf-life provided the results shown in Table 3. In this regard, *Salmonella* spp. and *L. monocytogenes* were absent in all the samples. Other pathogenic microorganisms, namely Enterobacteriaceae, *E. coli*, and *S. aureus*, showed <10 CFU/mL in all samples. These results support the safety for consumption of the beverages developed after fermentation and at the end of the storage period considered. The microbiological safety of these fermented beverages is favoured by low pH and high content of organic acids, in particular acetic acid, which inhibit the growth of most pathogenic microorganisms [38].

### 3.4. Qualitative and Quantitative Analysis of Anthocyanins by HPLC-DAD-ESI/MSn and HPLC-DAD

When profiling the anthocyanins present in the fermented drinks, it was worth noting that the main coloured phenolics detected in KWL were delphinidin-3-*O*-glucoside, petunidin-3-*O*-glucoside, peonidin-3-*O*-glucoside, and malvidin-3-*O*-glucoside in varying relative proportions to each other until the fourth day of fermentation. Nonetheless, the malvidin-3-*O*-glucoside content was the most significant contributor to the total anthocyanin content. Alternatively, in KGP, malvidin-3-*O*-glucoside was the only anthocyanin found at concentrations higher than the limit of quantification, being the most responsible compound for the anthocyanin burden throughout the entire study (Table A1). These results agree with those obtained by Costa-Pérez et al. [10] in a previous study that focused on the comprehensive characterisation of the (poly)phenolic profile of oenological by-products, where the highest anthocyanin content and the molecular diversity in wine lees relative to grape pomace were described.

**Figure 3:**
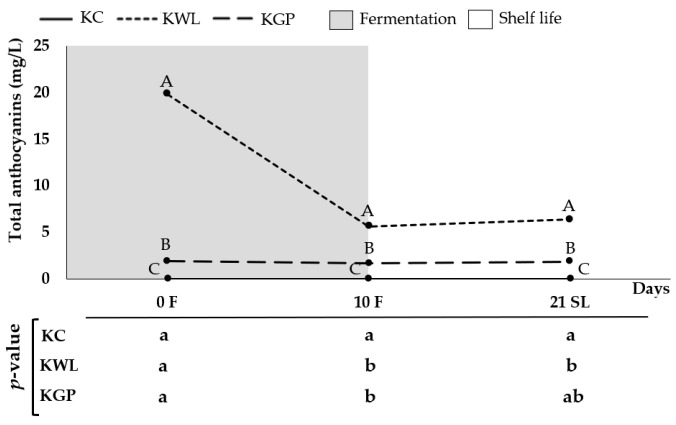
Total anthocyanin content (mg/L) at days 0 and 10 of fermentation and after 21 days of storage (shelf-life). Distinct lowercase letters indicate significant differences between fermentation days and shelf-life for same beverage, and distinct capital letters designate significant differences between beverages on same day. Statistical differences were retrieved by resorting to one-way analysis of variance (ANOVA) and Tukey’s multiple range test at significance level of *p* < 0.05. F: fermentation. SL, shelf-life; KC, kombucha control; KWL, kombucha wine less; KGP, kombucha grape pomace.

As expected, the kombucha-like beverages made by adding oenological by-products exhibited a higher anthocyanin content than KC, as illustrated in Figure 3. In particular, KWL showed an almost 10-fold higher concentration of these compounds than KGP, which is in line with what was observed in the initial infusions. Nonetheless, the concentration recorded in KWL decreased by approximately 72.0% during fermentation until reaching values of 5.60 mg/L. These results are in line with a previous study that demonstrated the decrease in anthocyanin content as a result of thermal- and microbially induced degradation pathways, active under prolonged fermentation conditions at 25 °C [11].

Despite this reduction, after finishing the development of the beverages, KWL continued providing almost a 3-fold higher concentration of anthocyanins than KGP (Figure 3), since in KGP, the effect of the fermentation on the anthocyanin content was less pronounced, with a decrease of only 11.0% (up to 1.68 mg/L at the end of fermentation).

The different efficiency as anthocyanin donors displayed by wine lees and grape pomace could be related to the internal source of these compounds in both materials. Thus, the anthocyanins provided by grape pomace release from grape skins [13], whereas in wine lees, more complex biological processes occur during wine fermentation as anthocyanins can be uptaken by yeasts. Thus, these compounds are absorbed by the mannoproteins that make up the outer surface of yeasts [39,40,41]. From this situation, during kombucha development, yeast’s autolysis leads to the release of the anthocyanins present in the cell wall into the medium, resulting in a high concentration of these compounds in KWL at the start of fermentation.

Once in the medium, anthocyanins were more exposed to degradation reactions in KWL because of the release of microbial enzymes (such as β-glucosidase) or the hydrolysis of these compounds by the enhanced microbiological population in comparison with the additional drinks assessed in the present work (e.g., LAB) [40,42,43]. However, these events can also affect KGP, as, in this type of beverage, the possibility of anthocyanins being taken up by the microorganisms that constitute the SCOBY is worth considering, as is the case with native yeasts present in the wine lees [40]. The main results further demonstrate the reverse relationship between fermentation time and anthocyanin content in kombucha-type beverages, as demonstrated for kombuchas prepared based on grape juice [32], pitaya by-product [2], Zijuan tea [7], among others. In contrast, the fact that the anthocyanin content of KGP decreased, to a lesser extent, during fermentation could be due to the anthocyanins’ interaction with other co-pigments, namely phenolic acids or flavonoids that are present in grape skins [17]. This phenomenon has been demonstrated to contribute to the protection of anthocyanins against microbial degradation during the fermentation process. For this interaction to occur, a molar ratio between anthocyanins and co-pigments must be established, which could have been saturated in KWL [42,44]. The association of anthocyanin stability and slowed down microbial metabolism is further supported by the fact that the concentration remained stable throughout the shelf-life of all beverages due to the effect of low storage temperatures (that slow down the microbial metabolism), which have previously been shown to preserve the stability of (poly)phenols for periods of up to four months in black tea kombucha [45].

### 3.5. Colour Analysis

**Figure 4:**
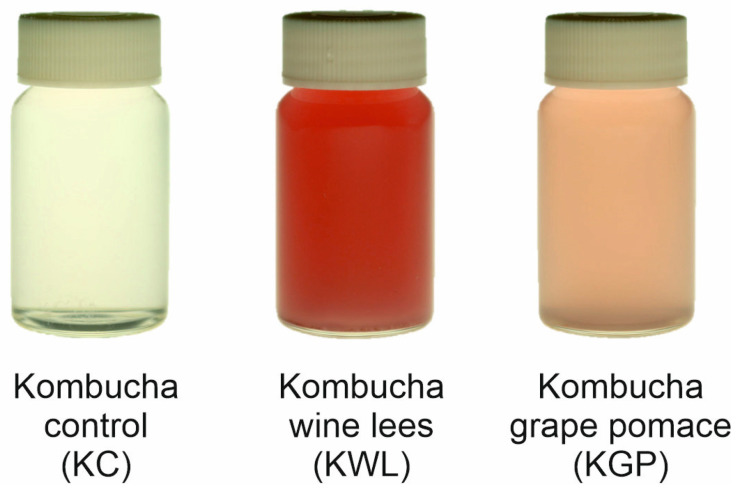
Different fermented beverages at day 21 of shelf-life.

Highly luminous red beverages were obtained. However, as expected, significant differences between KWL and KGP were observed in comparison with KC, in all the chromatic variables evaluated, as shown in Figure 4 and Table 4.

The beverages made from by-products, and more remarkably in the case of KWL, presented higher values in the parameters a*, b*, and Chroma, and lower values in L* and Hue angle. The parameter that showed the greatest divergence between the different beverages was the Hue angle, with values of 95.19, 32.90, and 18.58 at the end of the fermentation process for KC, KGP, and KWL, respectively. Since the Hue angle calculation is based on colour coordinates a* and b*, the beverages with a lower Hue angle showed a more reddish shade, represented by higher coordinates of a*, a condition highly valued by consumers [46]. Thus, the a* component of colour was also correlated to the anthocyanin content of the beverages, which shifts towards redder shades in acidic media, with the highest a* observed throughout the study in KWL, achieving values of 3.67 at the end of fermentation. Nevertheless, as presented in Table 4 and Figure 4, the chromatic characteristics of anthocyanins affected the beverages visually and, in most of the parameters evaluated, such as a decrease in lightness. This effect agrees with previous descriptions by Salar et al. in maqui beverages [20].

Significant differences in CIEL*a*b* colour space parameters were also observed during the fermentation process and shelf-life of beverages made from by-products. However, the colour difference (ΔE) of these beverages was less than 3, meaning these variations were not perceptible to the human eye [20].

### 3.6. Antioxidant Capacity of Kombucha-like Beverages

The antioxidant capacity of the beverages was determined using ABTS, FRAP, and ORAC assays, which provide complementary information depending on specific chemical reactions involved in maintaining the redox balance. These assays have been proven as particularly suitable for hydrophilic compounds such as (poly)phenols [47], which makes them especially useful. When comparing the radical scavenging capacity and reducing power of the kombucha-like beverages developed from oenological by-products with the control, grape pomace and wine lees provided drinks with significant antioxidant characteristics during the fermentation process and throughout shelf-life (Table 5).

The assessment of kombucha-like beverages based on wine lees and grape pomace infusions (KWL and KGP, respectively) regarding the evolution of the antioxidant capacity during development and storage showed that KWL significantly lost its antioxidant properties after fermentation, relative to the starting conditions by average percentages of 24.4% and 12.5% in the ABTS and ORAC assays, respectively, and by 10.7% concerning the reducing power (FRAP). Thereafter, it remained stable throughout its shelf-life. Alternatively, KGP antioxidant power did not vary significantly either after fermentation or at the end of the shelf-life monitored (Table 5). The invariable characteristic of KGP agrees with previous descriptions by Vukmanović et al. concerning the fermentation of kombucha made from the winery effluent [35].

Despite the different evolution of both types of drinks, KLW remained at the highest antioxidant level after each stage relative to KGP (Table 5). Specifically, KWL exhibited the highest antioxidant activity at the end of fermentation after storage, surpassing the values recorded for KGP by 16.1% and 23.2% in the ABTS and ORAC assays, respectively. In addition, the wine lees-based beverage exhibited a better reducing power compared with KGP, which displayed a 28.0% lower reducing capacity upon the FRAP test (Table 5). This trend remained almost constant during storage. At this stage, the KWL fermented infusions also exhibited the highest antioxidant activity and reducing capacity, while KGP rendered values between 16.1% and 21.2% lower (Table 5).

The improved antioxidant capacity recorded for KWL relative to KC and KGP fits properly with the also higher anthocyanin content, a phenolic class featured by a remarkable antioxidant power [46]. Thus, although grape pomace has been associated with a very valuable concentration of antioxidant compounds (e.g., tryptophan, melatonin, serotonin, and a range of (poly)phenolic classes) [10,47], the diversification of the (poly)phenolic profile seems to be in the base of enhanced radical scavenging and reducing powers of co-products developed from wine lees. Thereby, the different evolution of radical scavenging and reducing powers (Table 5) seems to be associated with the evolution in anthocyanin content (Figure 3), although mitigated by the presence of other bioactive compounds, especially in the wine lees, such as lipids, thiols, and sterols, which have been related to strong antioxidant properties [14,48]. In addition, the decrease in the phenolic compound burden is simultaneous with the production of new molecules, resulting from the metabolisation of the former, characterised by strong radical scavenging activity. The special relevance of these events in beverages developed using wine less, containing a diversity of metabolically active microorganisms [49], may suggest that these compounds would be generated as a result of microbial enzyme-induced changes in the structure of the phenolic compounds or by the interaction of the latter with other compounds, such as organic acids, which have been shown to exert a synergistic effect that increases their antioxidant activity [50]. In fact, in previous studies assessing the biological interest of white and red grape pomace-based kombuchas by Balmaseda et al. [9] and Barakat et al. [11], respectively, an enhancement in antioxidant capacity during fermentation was revealed. Thereby, the metabolic capacity not only of yeasts and bacteria in the SCOBY, but also the native yeast carried in wine lees, would be responsible for a specific profile of a higher value, for instance, providing sources of anthocyanin derivatives.

### 3.7. Global Overview of Changes in Quality Parameters of Kombucha-like Beverages by Multivariate Analysis

Summarising the main results concerning the (poly)phenolic burden and capacity to maintain the redox balance, KWL experienced the highest reduction, which would allow for hypothesising the central role of anthocyanins in the antioxidant activity of the kombucha-like beverages developed. To contrast this hypothesis, multivariate PCA and heatmap thermographic cluster analyses were performed.

**Figure 5:**
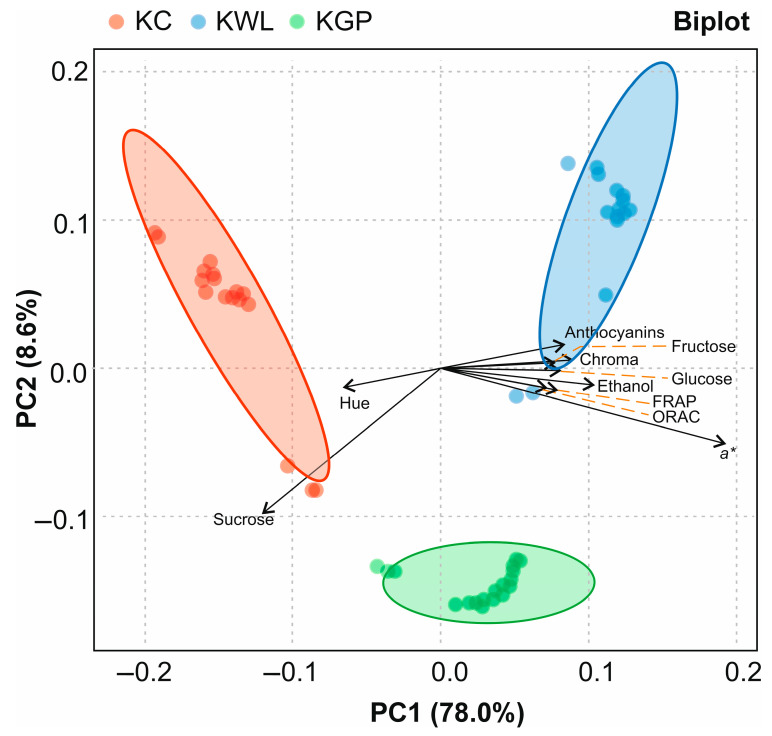
Principal Component Analysis (PCA) and eigenvectors biplot of all parameters analysed in fermented beverages. KC, kombucha control; KWL, kombucha wine less; KGP, kombucha grape pomace.

PCA grouped the obtained data into two principal components (PC1 and PC2), facilitating the identification of correlation patterns between variables. The PCA biplot in Figure 5 differentiated the clusters corresponding to the two beverages (KGP and KWL) and control samples (KC), using data from all the analyses performed. The first two principal components explained a high proportion of variability between samples (86.6%), allowing for reliable visual and statistical interpretation of sample differences. However, due to the differences obtained between the variations represented in PC1 (78.0%) and PC2 (8.6%), only the PC1 component was considered, since it explains the largest proportion of the total variability in the dataset. PC1 distinguished between the wine by-product beverages, which were clustered between the centre and the right side of the X-axis (upper-right quadrant for KGP and lower-right quadrant for KWL), and KC, which was clustered around in the negative section of the X-axis (Figure 5).

The values obtained in the by-product fermented beverages allowed for discriminating them from KC. Thus, KC was mainly distinguished by its higher Hue angle value and sucrose content relative to the experimental drinks, which are associated with a lower colour and fermentation rate, respectively [31,46]. On the other hand, KWL drinks were distributed in the upper-right quadrant, pulled specifically by the high values recorded for the anthocyanins, monosaccharides, and ethanol content, as well as the results from the ORAC and FRAP assays, and the colour parameters (Chroma and CIEa*, with the latter being especially relevant because of its association with the natural reddish colour of these beverages). Differences were also found between the two by-product beverages, with KPG distributed closer to the axis, with higher values for sucrose and Hue.

**Figure 6:**
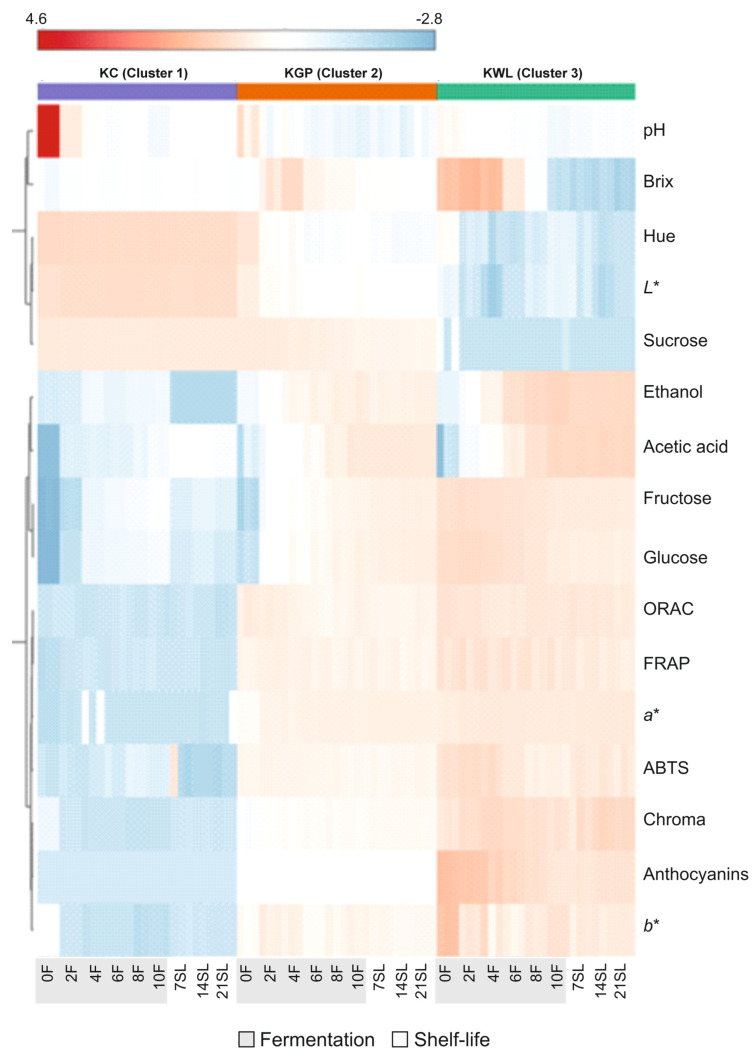
A heatmap graph of all the parameters analysed in different kombucha-like beverages during the fermentation process and shelf-life. The heatmap is a visualisation of the changes in abundance/level of features in rows for each beverage in columns. The colour ranges from deep red (high abundance or level) to deep blue (low abundance or level). F, fermentation; SL, shelf-life; KC, kombucha control; KWL, kombucha wine less; KGP, kombucha grape pomace.

The results of the PCA were consistent with those shown in the heatmap, in which a clear distinction between KC (Cluster 1) and the beverages produced using oenological by-products (Clusters 2 and 3) was confirmed (Figure 6). The latter showed the highest values for antioxidant activity, fermentation products, anthocyanin content, and certain colour parameters, such as Chroma, a*, and b*, which were represented by warmer colours. Additionally, the effects of the fermentation process and shelf-life included a higher initial content of most parameters, such as °Brix, glucose, fructose, antioxidant activity, and anthocyanin content, which decreased more markedly during the fermentation process in KWL. An increase in ethanol and acetic acid content was observed in beverages obtained upon fermenting winery by-product-based drinks. These traits result from the metabolism of the different microorganisms present in the SCOBY [31]. However, the metabolic consequences were more pronounced in KWL, which also underwent fermentations resulting from the metabolisms of native yeasts and bacteria.

The variations in the analysed parameters also showed relationships with each other obtained using Pearson’s correlation analysis. A positive correlation was also obtained between Chroma (colour intensity) and the FRAP-based reducing power (r^2^ = 0.820, *p* < 0.01), a relationship previously observed in beer and apple peel [51,52]. This relationship has been proposed as a consequence of the concentration and type of phenolic compounds present in the beverage, characterised by their chromatic properties and antioxidant capacity. This could also explain the positive relationship between anthocyanin content and the ORAC, ABTS, and FRAP antioxidant activity assays (r^2^ = 0.932, *p* < 0.01, r^2^ = 0.957, *p* < 0.01, and r^2^ = 0.914, *p* < 0.01, respectively), and negative correlation with the colourimetric parameter Hue angle (r^2^ = −0.723, *p* < 0.05) [20,47]. The correlation retrieved further supports the interest of valorising oenological residues as an ingredient source of bioactive (poly)phenols to obtain new high-quality and health-promoting foods.

## 4. Conclusions

The application of oenological by-products in the development of new “3S” fermented beverages was identified as a promising opportunity to use sustainable sources of bioactive compounds, such as anthocyanins, which are not found in traditional kombucha. This approach is especially relevant due to the biological attributes and health benefits associated with these coloured polyphenols. However, as expected, during fermentation and storage, the concentration of these compounds decreased significantly, remaining at levels previously described as health-promoting, thereby supporting the use of the assessed residues for the development of new fermented beverages. The relationship between anthocyanins and sugar concentration, as well as between the microbial community of beverages and the days of fermentation, is complex and can influence the stability of anthocyanins, constituting a critical issue to be addressed. From a future market perspective, the envisaged beverages, based on winery by-products, are safe and characterised by a naturally attractive red colour that will sustain the consumer’s choice, thus satisfying the first condition for achieving biological benefits, the selection and inclusion in dietary habits. Therefore, the present study opens up a new avenue for cross-sectoral innovation, merging fermentation traditions with the new organic and sustainable production of beverages characterised by differential nutritional and functional profiles, which will lead to the successful valorisation of oenological by-products and effectively advance towards circular economy in the food sector.

## Figures and Tables

**Table 1:** Total anthocyanin content (mg/kg dw) of infusions developed with differentially processed wine lees and grape pomace at distinct proportions.

Processed Oenological By-Product	Solid–Liquid Rate (g/L)	Total Anthocyanins
Oven-dried wine lees	20	218.89 ± 4.04 a B
	40	215.33 ± 4.65 a B
	60	147.20 ± 3.23 b B
Oven-dried grape pomace	20	16.16 ± 0.00 b D
	40	17.60 ± 0.16 a D
	60	13.33 ± 0.04 c D
Freeze-dried wine lees	20	378.28 ± 4.46 a A
	40	382.27 ± 4.83 a A
	60	281.43 ± 3.41 b A
Freeze-dried grape pomace	20	44.03 ± 0.81 a C
	40	33.17 ± 0.37 b C
	60	27.54 ± 0.86 c C

Data presented as mean ± SD (*n* = 3). Distinct lowercase letters indicate significant differences between infusions prepared by applying different solid–liquid rates within each matrix. Distinct capital letters indicate significant differences between infusions obtained using equal solid–liquid rates with diverse matrices. Statistical differences were retrieved by resorting to one-way analysis of variance (ANOVA) and Tukey’s multiple range test. Statistical differences were detected at statistical significance level of *p* < 0.05.

**Table 2:** pH, total soluble solids (°Brix), ethanol (mg/L), and acetic acid (g/L) measured at days 0 and 10 of fermentation and after 21 days of storage (shelf-life) in different beverages.

Parameter	Days	KC	KWL	KGP	LSD (*p* < 0.05)
pH	0 F	6.77 a A	3.64 a B	3.90 a B	0.25
	10 F	3.22 c A	3.19 c A	3.08 b B	0.06
	21 SL	3.52 b A	3.31 b B	3.27 b B	0.11
LSD (*p* < 0.05)		0.02	0.06	0.27	
°Brix	0 F	6.73 a B	7.76 a A	6.83 b B	0.11
	10 F	6.83 a B	6.25 b C	7.04 a A	0.11
	21 SL	6.87 a A	6.05 c B	6.89 b A	0.09
LSD (*p* < 0.05)		0.11	0.11	0.09	
Ethanol	0 F	18.00 b C	45.17 c B	84.33 b A	4.88
	10 F	75.00 a C	5593.33 a A	1363.50 a B	236.05
	21 SL	0.00 c C	4456.00 b A	1184.00 a B	167.75
LSD (*p* < 0.05)		6.21	177.77	228.57	
Acetic acid	0 F	0.00 c A	0.02 b A	0.06 b A	0.06
	10 F	0.21 b C	5.73 a A	2.55 a B	0.26
	21 SL	0.25 a C	5.84 a A	2.46 a B	0.13
LSD (*p* < 0.05)		0.02	0.25	0.14	

Data presented as mean (*n* = 3) with indication of Least Significant Difference (LSD) as dispersion indicator for significant differences at *p* < 0.05. Distinct lowercase letters indicate significant differences between fermentation days and shelf-life for beverage, and distinct capital letters indicate significant differences between beverages at same time-point. Statistical differences were retrieved by resorting to one-way analysis of variance (ANOVA) and Tukey’s multiple range test. F, fermentation; SL, shelf-life; KC, kombucha control; KWL, kombucha wine less; KGP, kombucha grape pomace.

**Table 3:** Analysis of pathogenic microorganism concentration (CFU/mL) in different fermented beverages produced.

Beverage	Days	*Salmonella* spp.	*Listeria* *monocytogenes*	Enterobacteriaceae	*Escherichia* *coli*	*Staphylococcus* *aureus*
KC	10 F	Absent	Absent	<10 ^Y^	<10	<10
	21 SL	Absent	Absent	<10	<10	<10
KWL	10 F	Absent	Absent	<10	<10	<10
	21 SL	Absent	Absent	<10	<10	<10
KGP	10 F	Absent	Absent	<10	<10	<10
	21 SL	Absent	Absent	<10	<10	<10

F, fermentation; SL, shelf-life; KC, kombucha control; KWL, kombucha wine less; KGP, kombucha grape pomace. ^Y^ Values below detection limit for Enterobacteriaceae, *Escherichia coli*, and *Staphylococcus aureus* (<10 CFU/mL).

**Table 4:** CIEL*a*b* values at days 0 and 10 of fermentation and day 21 of shelf-life in different kombucha-like fermented beverages.

Parameter	Days	KC	KWL	KGP	LSD (*p* < 0.05)
CIEL*	0 F	99.94 b A	97.50 a C	99.42 a B	0.21
	10 F	100.07 a A	97.28 a C	98.66 b B	0.14
	21 SL	100.07 a A	96.31 b C	98.46 c B	0.06
LSD (*p* < 0.05)		0.01	0.25	0.09	
CIEa*	0 F	−0.06 b B	2.43 c A	0.16 c B	0.36
	10 F	−0.01 a C	3.67 b A	1.32 b B	0.26
	21 SL	−0.01 a C	5.41 a A	1.54 a B	0.22
LSD (*p* < 0.05)		0.01	0.49	0.11	
CIEb*	0 F	0.42 a C	2.36 a A	0.67 b B	0.19
	10 F	0.13 c C	1.23 b A	0.85 a B	0.00
	21 SL	0.16 b C	1.01 b A	0.74 b B	0.01
LSD (*p* < 0.05)		0.01	0.18	0.09	
Chroma	0 F	0.43 a B	3.38 b A	0.69 b B	0.39
	10 F	0.13 c C	3.87 b A	1.57 a B	0.26
	21 SL	0.16 b C	5.50 a A	1.71 a B	0.21
LSD (*p* < 0.05)		0.01	0.49	0.14	
Hue angle	0 F	98.19 a A	44.32 a C	76.20 a B	2.14
	10 F	95.31 b A	18.58 b C	32.90 b B	1.91
	21 SL	92.40 c A	10.57 c C	25.66 c B	1.46
LSD (*p* < 0.05)		1.82	2.37	1.19	
ΔE	F	0.32 C	1.70 A	1.39 B	0.09
	SL	0.03 C	2.00 A	0.33 B	0.11

Data presented as mean (*n* = 3) with indication of Least Significant Difference (LSD) as dispersion indicator for significant differences at *p* < 0.05. Distinct lowercase letters indicate significant differences between fermentation days and shelf-life for beverage, and distinct capital letters indicate significant differences between beverages at same time-point. Statistical differences were retrieved by resorting to one-way analysis of variance (ANOVA) and Tukey’s multiple range test.F, fermentation. SL, shelf-life; KC, kombucha control; KWL, kombucha wine less; KGP, kombucha grape pomace.

**Table 5:** Radical scavenging activity and reducing power (mmol TE/L) at days 0 and 10 of fermentation and day 21 of shelf-life of kombucha-like fermented beverages.

Antioxidant Test	Days	KC	KWL	KGP	LSD (*p* < 0.05)
ABTS	0 F	0.26 b C	1.72 a A	1.16 a B	0.11
	10 F	0.37 a C	1.30 b A	1.09 a B	0.06
	21 SL	0.19 c C	1.46 ab A	1.15 a B	0.22
LSD (*p* < 0.05)		0.00	0.24	0.06	
FRAP	0 F	0.13 b C	2.72 a A	1.72 a B	0.34
	10 F	0.21 a C	2.43 ab A	1.75 a B	0.43
	21 SL	0.17 ab C	1.99 b A	1.67 a B	0.21
LSD (*p* < 0.05)		0.00	0.54	0.25	
ORAC	0 F	0.25 a C	2.81 a A	2.13 a B	0.29
	10 F	0.24 a C	2.46 b A	1.89 a B	0.11
	21 SL	0.20 a C	2.24 b A	1.86 a B	0.16
LSD (*p* < 0.05)		0.06	0.20	0.28	

Data presented as mean (*n* = 3) with indication of Least Significant Difference (LSD) as dispersion indicator for significant differences at *p* < 0.05. Distinct lowercase letters indicate significant differences between fermentation days and shelf-life for beverage, and distinct capital letters indicate significant differences between beverages at same time-point. Statistical differences were retrieved by resorting to one-way analysis of variance (ANOVA) and Tukey’s multiple range test. F, fermentation. SL, shelf-life; KC, kombucha control; KWL, kombucha wine less; KGP, kombucha grape pomace.

## Data Availability

The original contributions presented in the study are included in the article, further inquiries can be directed to the corresponding author.

## Abbreviations

The following abbreviations are used in this manuscript:

AAB	Acetic acid bacteria
F	Fermentation
SL	Shelf-life
KC	Kombucha control
KWL	Kombucha of wine less
KGP	Kombucha of grape pomace
LAB	Lactic acid bacteria
LSD	Least significant difference

## Appendix A

**Table A1:** Concentration of individual and total anthocyanins present in fermented beverages made from wine by-products.

Beverage	Day	Delphinidin 3-*O*-Glucoside	Petunidin 3-*O*-Glucoside	Peonidin 3-*O*-Glucoside	Malvidin 3-*O*-Glucoside	Total Anthocyanins
KWL	0 F	2.11 ± 0.25	3.08 ± 0.39	2.26 ± 0.23	12.35 ± 1.88	19.80 ± 1.53
	10 F	<LOQ	<LOQ	<LOQ	5.62 ± 0.04	5.62 ± 0.04
	21 SL	<LOQ	<LOQ	<LOQ	6.34 ± 0.07	6.34 ± 0.07
KGP	0 F	<LOQ	<LOQ	<LOQ	1.90 ± 0.04	1.90 ± 0.04
	10 F	<LOQ	<LOQ	<LOQ	1.69 ± 0.08	1.69 ± 0.08
	21 SL	<LOQ	<LOQ	<LOQ	1.82 ± 0.03	1.82 ± 0.03

LOQ, limit of quantification; F, fermentation; SL, shelf-life; KWL, kombucha wine less; KGP, kombucha grape pomace.
